# Qualitative evaluation of two London Faith and Health Networks: lessons learnt from a model of an interface between health systems and minority communities

**DOI:** 10.1136/bmjph-2024-001889

**Published:** 2025-04-05

**Authors:** Ana Zuriaga-Alvaro, Ben Kasstan-Dabush, Ella Johnson, Tracey Chantler, Leonora G Weil

**Affiliations:** 1 The NHSE Legacy and Health Equity Partnership, NHS England, London, England, UK; 2 The Vaccine Centre, Department of Global Health and Development, London School of Hygiene & Tropical Medicine, London, UK; 3 UK Health Security Agency, London, UK

**Keywords:** Trust, Public Health, Community Health, Vaccination, Qualitative Research

## Abstract

**Background:**

Ethnic and religious minorities in the UK had a higher risk of severe illness and mortality from COVID-19 in 2020–2021, yet were less likely to receive vaccinations. Two Faith Health Networks (FHNs) were established in London in 2022–2024 as a partnership approach to mitigate health inequalities among Muslim and Jewish Londoners through a health system–community collaboration. By evaluating the FHNs, this study aimed to examine: the organisational processes required for FHNs to serve as a model of interface between health systems and minority communities; the role these networks play in addressing public health inequalities; and implications for their future development and sustainability.

**Methods:**

A qualitative evaluation of the two FHNs was conducted using semi-structured interviews (n=19) with members of the ‘London Jewish Health Partnership’ and the ‘London Muslim Health Network’. Participant clusters included public health professionals, healthcare workers, community representatives and local government workers.

**Results:**

The FHNs shared similar structures of leadership, but differed in core membership, which influenced their access to expertise and the activities developed. They were found to perform a key conduit role by integrating expertise from within the health system and faith communities to address the needs and expectations of underserved communities, with the ultimate goal of addressing health inequalities through the design of tailored campaigns and services. Emerging themes for developing an FHN model included enhancing their sustainability by determining funding allocation, strategic integration into health systems and identifying the appropriate geographical scope to sustain their impact. Further implications included recognition of intersectionality, addressing diverse needs within faith communities and trust-building approaches.

**Conclusion:**

This evaluation offers insights into developing partnership models between faith-based organisations and health sectors to foster relationships with underserved communities. These findings provide valuable considerations for teams navigating the priority of health equity and community engagement as part of our learning from the pandemic to support the development of FHNs across different faith communities, not just for vaccine uptake, but to support the broader health and well-being of communities more widely.

**WHAT IS ALREADY KNOWN ON THIS TOPIC**

- Partnerships between faith-based organisations and health services take diverse forms, but the COVID-19 pandemic highlighted their potential to reduce health disparities by sharing accurate vaccine information and collaborating with health professionals on delivery strategies. Relatively less is known about how to build effective partnership models that can be sustained over time.

**WHAT THIS STUDY ADDS**

- This study evaluated two Faith Health Networks (FHNs) established in London, initially focused on COVID-19 efforts which later expanded to address broader health priorities. It offers insights into developing partnership models between faith-based organisations and health sectors to foster relationships with underserved communities and support their ownership over health delivery strategies.

**HOW THIS STUDY MIGHT AFFECT RESEARCH, PRACTICE OR POLICY**

- Future recommendations for developing FHN models include determining the ideal geographical scope of activities, responding to the diversity of faith groups, identifying where within the system they should be situated and ensuring strategic and sustainable funding. These models can offer long-term benefits as conduits between health systems and communities, which can be pivoted in pandemic preparedness and response efforts.

## Introduction

 The COVID-19 pandemic exposed and exacerbated existing health inequalities affecting ethnic and religious minorities in cities such as London.^1 2^ Evidence has indicated higher COVID-19 age-standardised mortality rates among Muslim and Jewish adults in England, and that certain communities were less likely to be vaccinated following the roll-out of the universal vaccine programme in December 2020.^3 4^ Faith and community organisations and leaders played an important role in supporting the COVID-19 pandemic response as ‘trusted messengers’ by sharing accurate and tailored health information around the efficacy and safety of COVID-19 vaccination and engaging with health professionals in developing appropriate delivery pathways.^5 6^ Partnerships established during the pandemic ranged from one-off events that hosted vaccination clinics in places of worship to more open-ended fora that addressed questions, concerns and misinformation. Partnerships between faith-based organisations and health sectors did exist prior to the pandemic,^7^ but health system leaders have increasingly recognised the need to involve partners in the design of health delivery strategies to reduce health disparities.^8 9^

**Figure 1:**
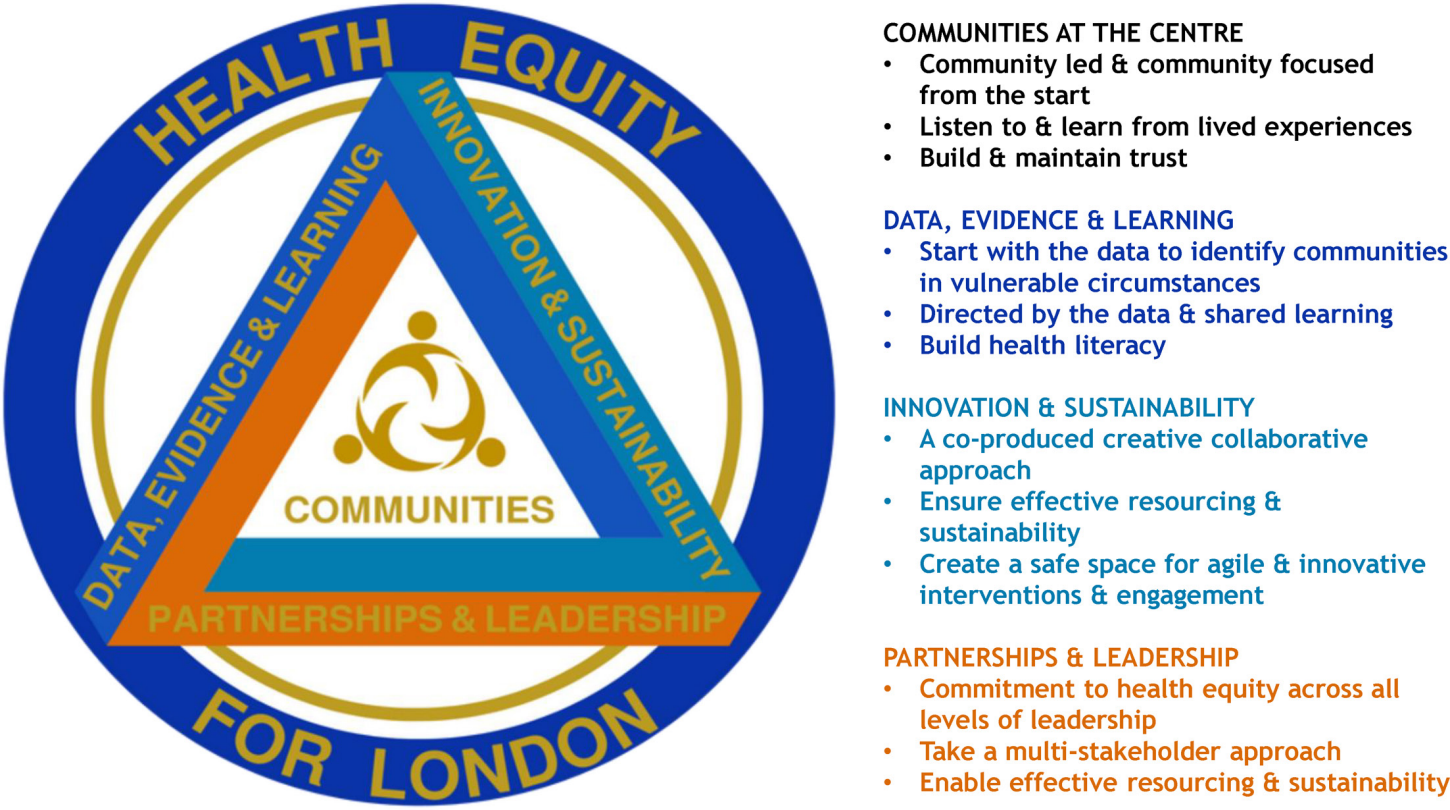
Processes, approaches and vision underpinning the ‘Legacy and Health Equity Partnership’ (LHEP) programmes of work, including the Faith Health Network models.

Public health leaders in London sought to build on lessons from the COVID-19 response by initiating the ‘Legacy and Health Equity Partnership’ (LHEP) as a 2-year multistakeholder programme (2022–2024), funded by the National Health Service (NHS) England.^10 11^ LHEP’s programme aimed to reduce inequalities in the provision of public health services for underserved populations through community-led collaborations. Priorities included improving rates of childhood immunisation coverage and adult cancer screening (e.g., breast cancer) by tailored engagement aimed at building trust and increasing the accessibility of NHS services.^11 12^ In early 2022, LHEP supported the development of two Faith Health Networks (FHNs); the London Muslim Health Network (LMHN) and the London Jewish Health Partnership (LJHP) that were formed on the request of representative organisations that serve Muslim and Jewish communities, following partnership working with health organisations during the pandemic. The underlying processes, approaches and vision underpinning the ‘Legacy and Health Equity Partnership’ (LHEP) and programmes of work including the FHN model are summarised in figure 1.

By evaluating the above FHNs, this study aimed to (1) consider the organisational processes required for a model of interface between health systems and minority communities; (2) examine the role these networks play in addressing public health inequalities; and (3) propose recommendations for their future contribution, development and sustainability.

### Background to the FHNs

During 2021, in response to emerging evidence of inequalities in COVID-19 vaccine uptake, London health partners brought together by the Greater London Authority facilitated a series of ‘Town Hall’ community events. These were online panel webinars with opportunities for questions and answers that included health experts and community leaders to support conversations on the COVID-19 vaccines to increase uptake and reduce vaccine hesitancy. These were developed with anchor organisations representing key communities of different faiths, ethnicities, ages and communities, particularly those with low vaccine uptake in the initial vaccine roll-out. Over 15 of these events were held, including one in March 2021 with the Jewish community, facilitated by the London Jewish Forum (LJF) and one in December 2021 with the Muslim community facilitated by the British Islamic Medical Association (BIMA). Following these events, representatives from these communities independently followed up with the organising health teams to request further partnership on the vaccine agenda, but also to support the health of people in their communities more generally, to act as a key conduit for effective co-produced engagement programmes to address community health needs and support outreach for key public health priorities.

LHEP was able to facilitate the development of these new networks with internal resources such as staff, programmtic expertise, funding for events and to establish governance arrangements for the networks within the wider health system to oversee and support the networks and to link them with relevant parts of the system as required. The intention was for these early networks to form ‘pilots’ for a model of partnerships of faith/communities with health partners on a regional footprint, beyond just the Muslim and Jewish communities. By early 2022, these networks were already starting to develop the network membership and terms of reference and successfully conducting their first community events, participating in Eid in the Square (May 2022) and the Maccabi Fun Run (June 2022). Further examples of activities led by both networks are included in online supplemental file 1.

Leadership of the two FHNs included a co-lead from the representative organisation (‘anchor organisation’ - the principal recipient of supplementary funding), and a Muslim/Jewish public health professional co-lead working in London health agencies. These two FHNs shared similar core objectives and leadership frameworks but varied in membership and activities. LHEP supported the secretariat in each FHN, providing operational support over the 2 years of the programme and facilitating links with relevant health partners across London including healthcare providers, public health agencies and other entities depending on the needs of the communities and FHNs. Initial activity centred on COVID-19, followed by routine vaccine uptake, subsequently broadening in scope to include other priorities (eg, mental health) as per community feedback. The anchor organisation supporting the LJHP was the LJF, which ‘advocates for the capital’s Jewish community, campaigning and influencing the public institutions affecting the lives of Jewish Londoners’.^13^ Members were drawn across the areas of London where most of the UK Jewish population is located, such as north central and northeast London boroughs of Barnet, Hackney, Camden and Haringey.^14^ Hackney is home to the largest Charedi (‘ultra-orthodox’) Jewish population in Europe.^15^ Building on past learning,^15^ LHEP recognised that ultra-orthodox (Charedi) Jewish communities required a focused approach to health communications and subsequently supported the development of the ‘Charedi Women’s Health Alliance’ as a subnetwork. Childhood immunisation campaigns were a key area of activity to help address the long-standing issue of suboptimal immunisation coverage in north London,^16^ and to support outbreak responses—such as the 2022 London Polio Booster campaign.^17^

The anchor organisation supporting the LMHN was BIMA, a voluntary organisation that ‘stands as a vibrant hub for the UK’s Muslim healthcare community, dedicated to engaging on health inequalities, advocating for equitable care, and building bridges between communities’.^18^ Muslims constitute 15% of the overall population of London,^19^ with communities forming large proportions of the population in Tower Hamlets (39.9%), Newham (38.8%) and Redbridge (31.1%).^20^ The LMHN concentrated its activities on pan-London community events and communication campaigns and usually established their meetings around them.

The LJHP and LMHN focused on developing tailored messages to engage their respective communities, and some approaches taken included co-designing and co-producing communications with community groups and lay people. Some of the messaging has involved religious–legal issues (eg, communications around porcine-derived immunisations), but most activities aimed to address health issues in ways that were relevant to each community by being sensitive to broader structural, systems and social factors that contribute to health inequalities. Building on learning from LJHP and LMHN, additional health networks were established to focus on inequalities among ethnic minorities, including the London Bangladeshi Health Partnership and INSPIRE for Black Londoners. These networks are outside the scope of this evaluation as they were less developed or established at the time of evaluation.

This evaluation was designed in response to the interest within London health organisations and communities to understand the value and impact of networks such as the LJHP and LMHN for reducing inequalities in underserved communities and how to sustain an FHN model beyond the 2-year core period of funding if they were found to be impactful.

## Methods

This study used a qualitative study design to evaluate the structure and organisation of the FHNs; data collection methods included semistructured interviews and review of FHNs reports and activities.

### Participant recruitment

Interview participants were identified for inclusion by each FHN co-chair and by TC, LGW, BK-D, AZA and EJ. Selection criteria sought to capture diverse standpoints of FHN members by including the professional roles, faith backgrounds, involvement in each FHN and through snowball sampling (see Results). The chairs of each FHN were consulted on the interview plans, as the membership and professional roles differed between networks. Potential participants were emailed a study information letter that included an invitation to take part in an interview. Emphasis was placed on the voluntary nature of participation and how the confidentiality of contributions would be maintained.

### Data collection and analysis

Data was collected by four interviewers TC, BK-D, AZA and EJ. All four had been involved in the FHNs to varying degrees, by attending network meetings and contributing insights or supporting the coordination of an FHN. Interviewers were assigned to an FHN they had less interaction with to minimise bias. Participants completed and signed a consent form before the start of the audio-recorded interview. Topic guides were used to steer the discussion and devised based on the terms of reference that underpinned the relevant network to reflect on their aims, objectives and priorities (see Consolidated criteria for Reporting Qualitative research). The interviews were conducted using digital software (Teams/Zoom) between October 2023 and February 2024 and ranged in length (20–60 min).

Audio recordings of the interviews were transcribed by the interviewers, and identifying information was removed from transcripts to protect the identities of participants. Data was analysed using inductive and deductive approaches.^21^ Each interviewer coded two interview transcripts each, and then met to develop a coding framework that reflected the emerging patterns as well as the overarching categories that informed the topic guide. The coding framework was updated iteratively, and the final categories and themes were discussed and defined in discussions with all the coauthors. Coding and the interpretation of data and comparison across categories and themes were conducted using Miro software.

### Public and patient involvement

LHEP hosted a public and patient involvement session in each network during September–October 2023 as part of the regular network meetings. These sessions aimed to explore how network members perceived the future of the FHN model beyond the core 2-year period of funding. The sessions discussed the role of the evaluation in supporting the potential of the FHNs and informed the development of topic guides.

## Results

The sample included 19 participants, 10 from the LJHP and 9 from the LMHN. LMHN participants were mainly healthcare providers (reflecting the make-up of this network), whereas LJHP participants were more diverse and included councillors, local authority and public health professionals and community leaders. All FHN co-chairs were interviewed (n=4). The data analysis identified three overarching themes: (1) Core elements of the LJHP and LMHN, (2) Purpose and goals of the LJHP and LMHN; and (3) Future development of the FHN model.

### Core elements of the LJHP and LMHN

Analysis revealed two core building blocks that characterised the LJHP and LMHN; membership and governance and leadership.

#### Membership

Membership of the LMHN was composed of multidisciplinary health professionals who identify as Muslim from a range of ethnic backgrounds (eg, British, Bangladeshi, Pakistani). LHEP funded a secretariat from the anchor organisation (BIMA), who had a professional background in community engagement and supported the network activities. Membership of the LJHP included representatives from: Jewish community organisations, Jewish healthcare professionals, elected representatives (eg, councillors), healthcare/community engagement professionals and academics serving Jewish populations. The multiagency and multistakeholder membership of the LJHP was considered crucial to drawing on the support of various regional and national health agencies when planning and implementing activities:

The multi-partnership is so important, that you’ve got NHS, Association of Directors of Public Health, you’ve got public health […] You know, we don’t do obesity and mental health in UKHSA. We don’t do that because we’re health protection, but then we can direct it into OHID [Office for Health Improvement and Disparities], into local authorities. Similarly, the NHS are going to be the experts in some things, but they wouldn’t be able to help with others. So, I think it is important that we go across our disciplines to the various different areas. (LJHP9)

Those interviewed reflected that the networks created a partnership space whereby health system and community representatives were brought together in a dedicated forum. This ‘health system-community approach’ that was adopted by both FHNs helped ensure that the needs and expectations of communities were adequately addressed by health organisations, and expertise of voluntary sector partners was leveraged in co-production activities:

Having professionals or people that have knowledge of that particular community is helpful. And having voluntary sector colleagues can provide some of that co-production co-design, but also then responding and being supportive to the needs of that community. (LMHN8)

By collaborating with local partners from the voluntary and community, or social enterprise (VCSE) sector, the FHN were able to build sustainable relationships with intended beneficiaries:

If you’re working with local partners that are supporting communities as part of their day jobs. That sort of legacy and longer-term sustainability of the initiatives are more likely to be there. (LJHP1)

Being a member of an FHN meant that learning around community engagement could be applied in their roles outside the networks to support broader place-based health delivery strategies:

The approach of the partnership has been to work on supporting the partners to do our jobs well, as opposed to trying to take over and become an independent organisation. So, in a sense, I say the partnership has enabled Hackney and Haringey public health teams, and [name of Jewish community group] to do our jobs more effectively*.* (LJHP6)

While there was a tendency to draw on community representation and membership from the VCSE, some reflected that an absence of lay membership prevented the inclusion of diverse voices and contributions:

Inevitably it is mainly through their representations within VCSE organisations. There’s probably not been too much in the way of ‘on the high street’ type engagement. (LJHP10)

#### Governance and leadership

By being under the auspices of LHEP and London health agencies, the FHNs were able to operationalise activities efficiently while not appearing as a ‘delivery arm for health agencies’:

It’s the fact that it’s a little bit detached from the system. I think that was the biggest strength, the fact that it sits within LHEP and that gave us a lot of connections and just made it easier to do some of the interventions that we wanted to do. (LMHN2)

The co-chair leadership approach whereby one chair came from a community faith-based ‘anchor’ organisation and the other was a public health professional of Jewish or Muslim heritage was viewed as essential:

The other thing is, which I think was very important with the networks, is the leadership had to be from the communities. I think that is really important […] all of the networks are co-chaired by the community, but one is more closely linked with the community and generally one more is closely linked with the health board. (LJHP9)

The networks were ‘volunteer-driven’ and participants in both FHNs highlighted the challenge of relying on the goodwill of the members, especially those (eg, chairs) responsible for coordination and delivering action points:

Whether there’s capacity really, because then, again, you’d be looking at everybody’s kind of giving up their time, I guess, in a voluntary capacity*.* (LMHN3)

### Purpose and goals of the LJHP and LMHN

Participants suggested that the purpose of their FHN was to act as a conduit between the faith communities they serve and the health system, which facilitated the goal of developing tailored communication and engagement approaches:

That was really around being a conduit between health and care and communities, and designing and developing sort of culturally appropriate and faith specific engagement opportunities that addressed the really key health challenge. (LMHN1)

The ability to produce community-specific communications and learn from feedback helped to refine engagement approaches and foster trust with intended beneficiaries of the FHN (figures1  2):

I think a lot of it is also around trust as well and engendering trust and to understand some of the issues and gain that intelligence from communities in terms of what people are thinking, how they’re consuming messages and what things are landing and not landing as well*.* (LMHN5)

**Figure 2:**
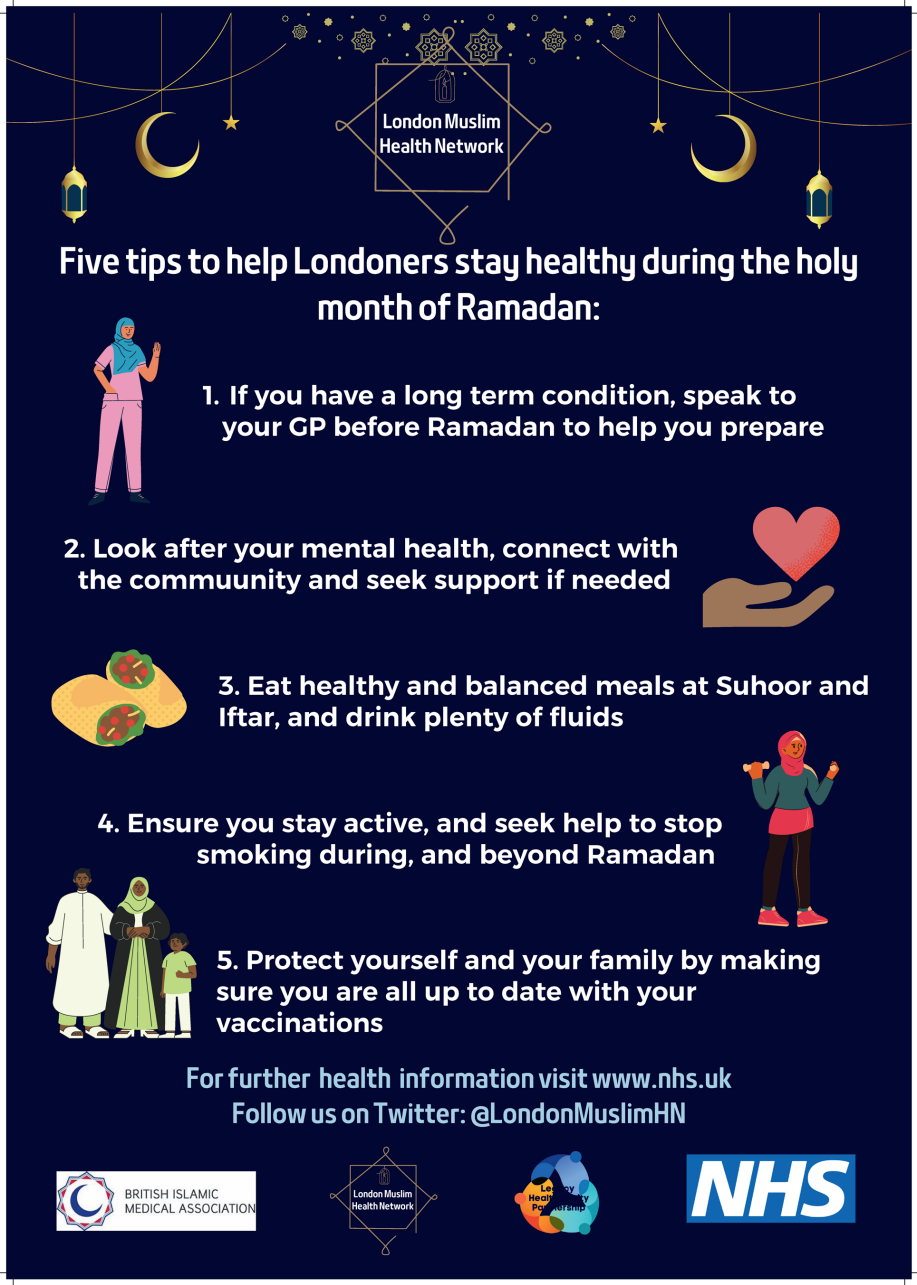
London Muslim Health Network communication, 2023. GP, general practitioner; NHS, National Health Service.

Building trust through community representation and partnerships was pivotal to the longer-term goal of reducing health inequalities through more accessible services and tailored information:

That’s where I think our interventions being more specific and being culturally sensitive and specific have enabled people to understand and receive personalized care and health advice. So, I think that is a is a major step in trying to reduce health inequalities to making it more accessible but also I think there’s something about you know, actually having health professionals who are Muslim leaders and part of the community*.* (LMHN8)

FHN goals were to undertake community engagement in advance of key religious festivals (eg, Eid; Chanukah) and in a timely manner due to public health incidents, such as the spread of polio, pertussis and measles:

Communications has been linked to sort of particular Jewish holidays as well. So, the timing of communication to take into account particular celebrations appropriate messages for the celebrations. (LJHP6)

This approach was considered to diverge from past attempts to deliver health information. The main difference being that FHN provided the opportunity to shape communications in a ‘culturally sensitive’ manner (see figures2  3):

We had feedback [that] communications were very culturally sensitive, different from how they had not always been in the past, which was valuable*.* (LJHP6)

**Figure 3:**
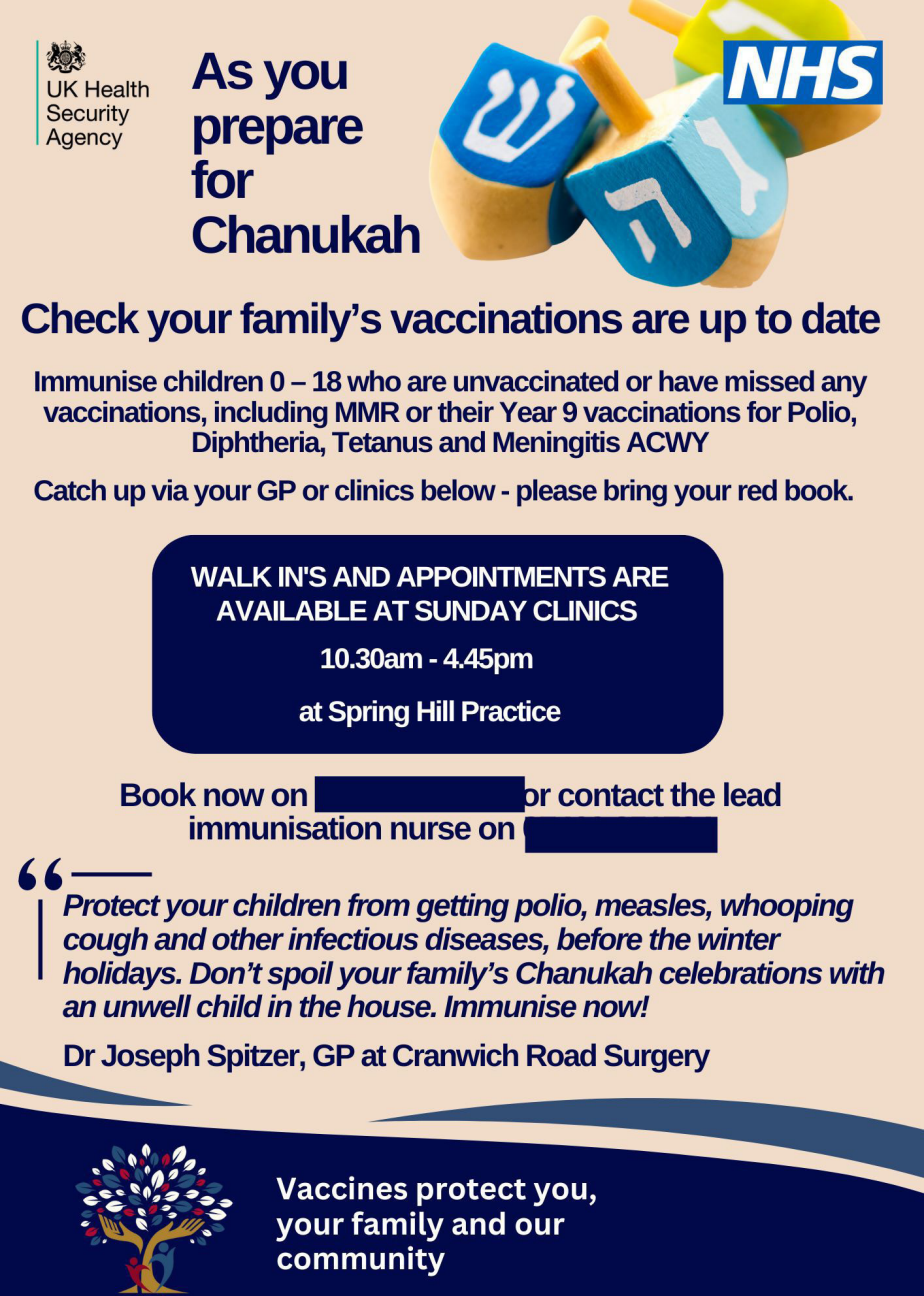
London Jewish Health Partnership communication, 2023. GP, general practitioner; MMR, measles, mumps and rubella; NHS, National Health Service.

Participants noted the need to increase the public visibility of the FHNs because there were no established ways for intended beneficiaries to access information about the FHNs, provide feedback and get involved (eg, a website):

It’s one of the most common questions that we got asked when we did community engagement opportunities was, ‘how do I get involved?’ from Muslim health professionals so there was a real appetite from wherever we went people were excited to see something happening addressing health and health inequalities specifically within Muslim communities. (LMHN1)

Some of those interviewed did not view the lack of public visibility as an issue because it was seen that their main goal was for intended beneficiaries to receive tailored health messages even if it was not apparent who had developed them:

I don’t think the public need to see everything behind the closed door, but I would like to hope that they see the adverts, even if they don’t know who did them […] I think it’s a bit like a kind of public health team. The community don’t need to know, but their health is being managed by this team. (LJHP9)

Feedback reflected the need to recognise that faith communities are not homogeneous—that there is diversity within organisations and that there could be intersectionality across groups and the needs of the networks to recognise this:

I think whether it’s the Jewish health network or whether it’s the Muslim health network, I think people often ‘say let’s go and engage with the Muslim community, let’s go and engage with the Jewish community’. Actually, it’s far more complicated though, there’s other intersections like cultural backgrounds, there’s particular sort of sects and nominations within Muslim communities that sort of that might not be represented. So, did it have full reach within the Muslim community? No, I think it didn’t. (LMHN8)

### Future development of the FHN model

Recommendations for future development of FHN models coalesced around the need for allocation of consistent funding to ensure that FHNs were sustainable. This was seen to be important to maximise the added value of FHNs and their ‘on the ground’ delivery approach that enables dedicated engagement with underserved communities that is essential for addressing health inequalities:

I think there has to be funding available for these networks to continue, whether that means having a project manager and a team behind actually making sure that these health networks go ahead, that they’re constantly evolving and they’re moving and they’ve got agenda going forward. I think the delivery element is really important. Not delivery in terms of so vaccines in arms, but generally in terms of engagement with the public, and on the ground engagement, I think it’s really important. I think that the strategic element of it is, and things like decision making and influencing systems is important, too. But I think it’s important not to lose that delivery element of the faith networks as well*.* (LMHN3)

Questions were raised around the most strategic place for the FHN model to be positioned, whether within the health system or independent from it and the need for this to be considered in connection to the funding models:

I personally think it needs to sit somewhere close to the system, there needs to be an oversight or there needs to be almost accountability from the system to support these faith networks. Because, yes, we can go ahead and start applying for funds. So, work with certain communities, like with certain councils or ICBs [NHS Integrated Care Boards], to get some funding for certain events, I think that can happen. But unless there is a system accountability for these growth networks, how can we support them, be it financial, but also political push for these networks to carry on and to be established? (LMHN2)

Participants differed in their views of the appropriate geographical remit of FHNs, and whether this should be at borough, integrated care board, NHS region or national level. The NHS London regional scope of the LJHP and LMHN was viewed as a strength by some, as it allowed rapid and agile responses (as in the London polio booster campaign) by drawing on resources intended for a regional remit:

Jewish communities are all across London. It just meant we could pull on regional resources, thoughts, ideas and tie up with some of the priorities and get that kind of two-way conversation between what was going on from a regional footprint, what was happening hyper-locally, spread it with different regions. (LJHP9)

On the other hand, looking beyond regional limits was pragmatic for communities that are connected beyond defined borders:

I’m not a fan of the way we use geography as a way of actually kind of thinking that how communities interact. So, I think there is something about what’s the best thing is depending on you know almost kind of going down the ethnographic route to being observing how people use spaces where they go and how they go and that should kind of give you a better sense of where they go (LMHN6)

Participants stated that they would value the opportunity for collaboration and exchange across health networks to share best practice, explore different methods of working and contribute to collective growth:

INSPIRE [community health network for Black Londoners, see Introduction] has been really keen to learn from the experiences of the Jewish network partnership, and the Muslim network. (LJHP9)

It would be interesting to understand the ways of working for different networks. Different networks obviously operate in different ways. And we’ve seen a lot of impact coming in through project delivery. So, it’d be good to understand is there a particular frame that they want the networks to operate? Is it a case that they will have set targets with networks? I mean, we’ve just been delivering how we see fit of what’s needed for the community. (LMHN9)

This suggests that the LJHP and LMHN were viewed as an asset to support community health, but that members saw a benefit from learning how different networks and models of engagement between faith and communities and health systems had developed.

## Discussion

This evaluation of the LJHP and LMHN highlighted three overarching themes: first, the core elements required for developing a network included the multi-disciplinary membership and governance; second, the purpose and goals highlighting the aim to bridge the gap between faith communities and the health system and providing culturally sensitive engagement to address health inequalities; and finally, considerations for future development emphasising the need for consistent funding, strategic integration with health systems and regional collaboration to sustain their impact and expand reach. Several important aspects emerged, particularly around intersectionality, with a need to address specific needs of different groups within these faith communities to ensure inclusive and representative outreach. Developing communications that were accurate, yet sensitive and tailored, especially around religious holidays or localised outbreaks, were perceived by FHN members to make health information more accessible and acceptable to their communities and hence engender trust in health services. Key challenges highlighted included ensuring diversity within community representation and addressing the long-term sustainability of these initiatives.

The FHNs were established to address vaccine inequity and broader health inequalities by engaging and building sustainable relationships with faith communities. The LJHP and LMHN shared similar structures in terms of organisation and co-leadership but differed in core membership. The latter was formed primarily of Muslim healthcare professionals and in contrast with the LJHP did not include representation from VCSE and local authority public health teams. The differences in membership influenced the events and activities that each FHN organised (see online supplemental file 1). The FHNs performed a key conduit role by integrating expertise from within the health system and faith communities to develop campaigns and activities that helped to address the needs and expectations of communities. Those interviewed reflected on the importance of these networks in developing longer-term, sustainable relationships with underserved communities. Against the backdrop of the disproportionate burden of the COVID-19 pandemic experienced by underserved minorities in London,^1^,^3^ both Jewish and Muslim communities faced unique and significant challenges. For instance, higher mortality rates were recorded among both groups,^4^ while lower vaccine acceptance was particularly notable among Muslims and strictly Orthodox Jewish communities.^1 3^ Moreover, strictly Orthodox Jewish communities have anecdotally reported elevated rates of infection and hospitalisation.^22^ These disparities can be attributed to a combination of socioeconomic and cultural factors, such as large families in Jewish communities and multigenerational family homes among South Asian communities,^23^ occupational exposures, pre-existing health inequalities and cultural and religious practices, such as communal prayers and rituals, presented distinct vulnerabilities that required tailored public health guidance. These challenges underscored the critical need for co-produced culturally sensitive health messaging and interventions, which FHNs were instrumental in delivering. The ability of the FHN to foster relationships was recognised as a priority within resource-constrained environments.

Partnerships between faith-based organisations and healthcare systems are not a new phenomenon and can take diverse forms, but the COVID-19 pandemic placed renewed attention on faith and health as a key policy concern in the UK and internationally.^79 24^,^26^ The WHO developed the ‘WHO Faith Network’ during the COVID-19 pandemic, as a strategy to increase access to accurate information but also supporting healthcare delivery strategies by developing toolkits on engaging faith leaders and communities during health emergencies.^27^ In the UK, the National Institute for Health and Care Research made funding available in 2023 for researchers to assess the question of ‘How can engagement with faith-based groups impact health and health inequalities?’.^28^ In 2023 the UK Health Security Agency National Conference included a timetabled session focused on faith in health protection.^29^ In December 2023, over 100 attendees from community organisations and lay backgrounds joined a London faith and health conference.^30^ Building on the emerging interest in faith and health, the results presented in this qualitative evaluation offer insights into how partnership models can be developed and scaled up.

This evaluation highlights key considerations for the development of future FHN models in different communities. Public visibility and transparency of the organisational structure and membership of FHNs was flagged as a priority for future models. A key suggestion was for future FHN models to include ‘lay people’ (in addition to representatives from community organisations) in their core membership to ensure that they provide input on strategic goals. Their involvement would also allow people who have personal experience of using health services to participate in co-producing communication materials and activities. Public health researchers have defined ‘co-production’ as ‘Collaboration between community members, researchers and policy makers drives efforts to solve complex health problems’.^30^ Co-production is an established practice in public health and builds on a long trajectory that emphasises the importance of citizens being given responsibility to shape the services that they are offered.^31^ Agreeing on the methods and scope of co-production in FHNs’ terms of reference may help to ensure accountability to the communities being served. The volunteer model of the FHN was not considered sustainable, and dedicated project management is required for the networks to achieve their goals.

Another key consideration regards the ‘footprint’ of the network, whether they have a regional scope or how to accommodate how faith communities live across administrative boundaries.^32^ The sustainability of FHNs will depend on how they are integrated within the health system infrastructure and remain accountable to the communities they represent. Allocation of funding by the healthcare system brings a need for partnership models to demonstrate impact on health equity, which is not straightforward.^33^ For example, it was difficult to disaggregate the impact of FHN activities on vaccine uptake amidst the 2022 London polio booster campaign due to the simultaneous efforts across the health system. However, we argue that models of community engagement are valuable as they create forums for underserved communities to take ownership of how healthcare is delivered. FHN can then offer a longer-term benefit by serving as a ‘trusted’ partner in community health that can be pivoted in pandemic preparedness and response efforts. Lastly, commissioners will need to recognise that the partnership models of FHNs will vary according to the degree of institutional representation of communities. Roma, Gypsy and Traveller communities, for example, experience profound social and health inequalities but differ in institutional representation, which will shape approaches to co-design and engagement.^34^

### Strengths and limitations

A strength of the evaluation was a consideration of the core elements and building blocks of an FHN partnership model amidst increasing interest and investment in the field of faith and health. One key limitation is that all coauthors had been involved in the FHNs to varying degrees, which could have contributed to bias during data collection and analysis. Another limitation was that evaluation activities were not embedded in the design and implementation of the FHNs, which meant there was less opportunity to observe activities and document lay perceptions of their relevance. Future studies should explore the lessons from FHN networks that apply to broader community health groups such as those focused on ethnicity or geographical location.

## Conclusion

Disparities documented during the COVID-19 pandemic signalled the imperative of establishing frameworks for sustained and trusted engagement with underserved communities. This evaluation suggests that FHNs are a pathway to nurturing that relationship. The LJHP and LMHN offer examples of the organisational processes that are required to build and sustain an FHN to best facilitate constructive collaboration between health agencies and faith communities, with an ultimate goal of reducing health inequalities. The partnership models of FHN will vary according to the institutional representation of communities, and these lessons can be applied beyond the Muslim and Jewish networks to support the development of FHNs across different faith communities, not just for vaccine uptake but to support the broader health and well-being of communities more widely.

## Supplementary material

10.1136/bmjph-2024-001889 online supplemental file 1

## Data Availability

All data relevant to the study are included in the article or uploaded as supplementary information.
