# Supplementary material for: Qualitative evaluation of two London Faith and Health Networks: lessons learnt from a model of an interface between health systems and minority communities
Source: BMJ Public Health. 2025 Apr 5;3(1):e001889. doi: 10.1136/bmjph-2024-001889 (PMC11973757; doi:10.1136/bmjph-2024-001889)
Supplement: online supplemental file 1 [file bmjph-3-1-s001.docx]

**Supplementary file 1: Activities delivered during the first two years of the networks (2022-2024)**

| **The London Jewish Health Partnership** | | |  |
| --- | --- | --- | --- |
| **Activity** | **Description** | **Value** | **Some examples and dates** |
| **Regular meetings** | The LJHP aimed to establish 5-6 regular meetings per year, with additional meetings scheduled as needed for specific activities such as preparing communication campaigns and organizing health stands. | Meetings included a brief period for standing agenda items, such as partner updates and action progress, with the majority of the time focused on key deliverables and tasks. This structure ensured that routine updates are covered efficiently, allowing sufficient time for collaborative work on primary objectives. | The first scoping meeting was held in May 2021. Meetings focussed on topics ranging from vaccination, screening and mental health |
| **Deep dive workshops** | The network organised deep-dive workshops of topics consensually agreed upon by its members (e.g., immunisations, screening, mental health) | Specialists, health professionals, leaders, and interested partners working with and from the Jewish community were invited to themed deep-dive workshops. These workshops aimed to leverage their expertise to understand challenges and collaboratively develop creative and practical solutions for regional and local implementation | -Immunisation Deep Dive Workshop in partnership with the London Jewish Community (online session): September 2022  -Focus Session on Polio and other Childhood Immunisations (online session): March 2023 |
| **Health stands** | Health stands at community events co-organised and designed by LJHP and local, regional and national health partners: the Maccabi Fun Run at StoneX stadium (Barnet, London) and the Shomrim event in Stamford Hill (Hackney, London) | These were family events that provided a valuable opportunity to engage with the community by offering tailored health information on topics like childhood immunisations, cancer screening, oral health and mental health. The events also improved access to healthcare services, such as free blood pressure checks, and facilitated direct conversations with health professionals. This visible presence in the community helped build trust and foster relationships between the community and the broader health system | -Maccabi Fun Run event:   - June 2022 - June 2023 - June 2024   -Shomrim event (sub group Charedi Women's Health Alliance): July 2023  -Menucha Big Talk event (sub group Charedi Women's Health Alliance): May 2024 |
| **Communication campaigns** | Various communications and campaigns based on health issues affecting the community, including threat of Polio, measles and pertussis. | These campaigns aimed to enhance community motivation to vaccinate, personally and for children, through collaboration between NHS and local communities for culturally competent campaigns raising awareness of symptoms, susceptibility, and severity. The LJHP partnered with trusted voices and media for vaccine information dissemination. | -Polio campaign: September-2022  -Communications and engagement campaign for increasing childhood immunisations uptake in London Jewish communities: A community and health system partners partnership: December 2023 |

| **The London Muslim Health Network** | | |  |
| --- | --- | --- | --- |
| **Activity** | **Description** | **Value** | **Some examples and dates** |
| **Regular meetings** | The LMHN aimed to schedule 4-6 network meetings a year. Additionally, the network held more frequent meetings in the lead-up to key community engagement events. | These regular meetings provided consistent opportunities for collaboration, planning, and addressing urgent needs, ensuring effective preparation and execution of community events. | The first scoping meeting was held in early 2022 and early planning focused on preparations for the first Eid in the Square event. |
| **Health stands** | Health stands at community events led by the LMNH with support from regional and local health partners and volunteers. Eid in the Square at Trafalgar Square (Westminster, London), the Halal food festival at London Stadium (Hackney, London), Health Awareness Stand at the London Muslim Shopping Festival (Tower Hamlets, London) | These events were mainly attended by families and the aim was to engage with members of the Muslim community regarding specific areas of health inequalities including long-term conditions, diabetes, hypertension, mental health, and immunisations. Volunteers supporting the stands were largely from Muslim faith communities themselves, and many spoke community languages. The stands were offering on-site blood pressure and blood sugar checks, along with wider MECC health and wellbeing conversations. | -Eid in the Square:   - May 2022 - April 2023 - April 2024   -London Halal Food festival   - September 2022 - September 2023   -London Muslim Shopping Festival: February 2024 |
| **Activities in Mosques** | The Women's Breast Cancer event and the Nutritional Ramadan cooking workshop (diabetes cooking workshop) at Ar-Rahman Mosque and the East London Mosque Pop-up vaccination clinic statistic | These activities were delivered directly within the community, offering tailored health education, practical resources, and accessible healthcare services, enhancing community engagement and support for effectively managing health concerns. |  |
| **Communication campaigns** | Various communications and campaigns addressed health issues affecting the community, focusing on topics such as childhood immunizations, breast screening, and health considerations during Ramadan. | These communications enhanced health promotion, improved accessibility to vital information, and encouraged proactive health management in a culturally resonant manner. For example, using traditional bread-making techniques to teach self-examination for early signs of breast cancer. | -Health considerations campaigns during Ramadan: April 2023-2024 |
